# Exploring Radiographic Progression-Free Survival in Diverse Subgroups of Metastatic Hormone-Sensitive Prostate Cancer: Comparative Efficacy of Abiraterone and Enzalutamide

**DOI:** 10.3390/jcm15135012

**Published:** 2026-06-27

**Authors:** Aykut Özmen, Deniz Tural

**Affiliations:** 1 Department of Medical Oncology, Başakşehir Çam and Sakura City Hospital, Istanbul 34480, Turkey; 2 Department of Medical Oncology, Koç University Hospital, Istanbul 34010, Turkey; deniztural@gmail.com

**Keywords:** metastatic hormone-sensitive prostate cancer, abiraterone, enzalutamide, androgen receptor pathway inhibitors, radiographic progression-free survival, real-world study, subgroup analysis

## Abstract

**Background/Objectives:** Metastatic hormone-sensitive prostate cancer (mHSPC) is a biologically heterogeneous disease in which treatment intensification with androgen receptor pathway inhibitors has significantly improved clinical outcomes. However, direct comparative evidence between abiraterone and enzalutamide remains limited. We aimed to evaluate radiographic progression-free survival (rPFS) in patients with mHSPC treated with first-line abiraterone or enzalutamide and to perform exploratory subgroup analyses according to baseline clinical and laboratory characteristics. **Methods:** This retrospective single-center study included patients with mHSPC who received first-line abiraterone or enzalutamide in combination with androgen deprivation therapy. Baseline demographic, clinical, and laboratory characteristics were collected retrospectively. The primary endpoint was rPFS. Survival was analyzed using the Kaplan–Meier method and compared using the log-rank test. **Results:** A total of 172 patients were included, of whom 84 received abiraterone and 88 received enzalutamide. The median follow-up duration was 24.7 months (95% CI 21.5–27.9). In the overall population, median rPFS was comparable between the abiraterone and enzalutamide groups (50 vs. 49 months, *p* = 0.21). However, enzalutamide was associated with significantly longer rPFS among patients aged <70 years (HR 0.25, 95% CI 0.07–0.89; *p* = 0.02), those with baseline hemoglobin ≥12 g/dL (HR 0.36, 95% CI 0.15–0.85; *p* = 0.01), and those with baseline ALP < 147 U/L (HR 0.43, 95% CI 0.19–0.98; *p* = 0.04). No significant differences were observed in other subgroups. **Conclusions:** rPFS was comparable between abiraterone and enzalutamide in the overall mHSPC population. However, enzalutamide was associated with longer rPFS in patients aged <70 years and in those with preserved hemoglobin and lower ALP levels. These findings suggest that baseline clinical and laboratory characteristics may influence treatment outcomes and should be prospectively validated.

## 1. Introduction

Prostate cancer remains one of the most commonly diagnosed malignancies and a leading cause of cancer-related mortality among men worldwide. Metastatic hormone-sensitive prostate cancer (mHSPC) represents a biologically heterogeneous disease with highly variable clinical outcomes despite substantial therapeutic advances over the last decade. Historically, androgen deprivation therapy (ADT) alone constituted the standard treatment for patients with newly diagnosed metastatic disease. However, multiple randomized clinical trials have demonstrated that treatment intensification with systemic agents significantly improves survival outcomes compared with ADT alone, leading to a paradigm shift in the management of mHSPC [1,2,3,4].

Among the available treatment intensification strategies, androgen receptor pathway inhibitors (ARPIs), including abiraterone acetate and enzalutamide, have become widely adopted first-line treatment options. Abiraterone demonstrated significant improvements in overall survival and progression-free survival in both the LATITUDE and STAMPEDE trials, while enzalutamide showed substantial clinical benefit in the ENZAMET and ARCHES studies [1,2,3,4]. As a result, contemporary international guidelines recommend both agents as standard-of-care treatment options for patients with mHSPC [5]. Despite the established efficacy of these therapies, selecting the optimal ARPI for an individual patient remains challenging in routine clinical practice. Unlike randomized clinical trials, real-world treatment decisions are frequently influenced by patient age, comorbidities, disease burden, laboratory parameters, physician experience, treatment accessibility, and potential toxicity profiles. Furthermore, prospective head-to-head randomized comparisons between abiraterone and enzalutamide are currently lacking, and the available comparative evidence is largely derived from indirect comparisons and studies conducted in metastatic castration-resistant prostate cancer populations [6,7].

In addition to treatment selection, considerable interest has emerged regarding the prognostic role of baseline clinical and laboratory characteristics in advanced prostate cancer. Factors such as performance status, metastatic burden, visceral involvement, hemoglobin (Hgb) level, prostate-specific antigen (PSA), alkaline phosphatase (ALP), and lactate dehydrogenase (LDH) have consistently been associated with clinical outcomes in metastatic prostate cancer and other advanced cancer settings [8,9,10,11]. These variables are routinely available in clinical practice and may help identify patient subgroups with distinct disease biology and treatment responsiveness. Although prognostic factors have been extensively studied in metastatic prostate cancer, data evaluating whether commonly used baseline clinical and laboratory parameters may be associated with differential outcomes between specific ARPIs in the mHSPC setting remain limited. Improved understanding of these factors could potentially contribute to more individualized treatment selection and generate hypotheses for future prospective investigations.

Therefore, in the present study, we aimed to evaluate radiographic progression-free survival (rPFS) outcomes in patients with mHSPC treated with first-line abiraterone or enzalutamide in a real-world setting, with particular focus on exploratory subgroup analyses according to baseline clinical and laboratory characteristics.

## 2. Materials and Methods

### 2.1. Study Design and Patients

**Figure 1:**
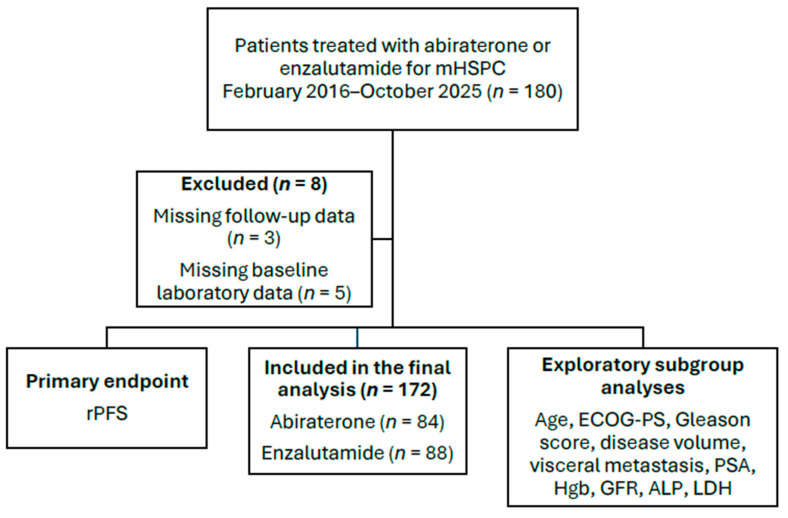
Study flowchart (ALP: Alkaline phosphatase, ECOG-PS: Eastern Cooperative Oncology Group Performance Status, GFR: Glomerular filtration rate, Hgb: Hemoglobin, LDH: Lactate dehydrogenase, mHSPC: Metastatic hormone-sensitive prostate cancer, PSA: Prostate specific antigen, rPFS: Radiographic progression-free survival).

This retrospective study included patients with mHSPC who received first-line ARPI treatment with either abiraterone acetate or enzalutamide in combination with ADT at our institution between February 2016 and October 2025. Patients were identified through institutional electronic medical records. Eligible patients were required to have histologically confirmed adenocarcinoma of the prostate, radiologically confirmed metastatic disease, and hormone-sensitive status at the initiation of ARPI treatment. Patients who received abiraterone acetate plus prednisone or enzalutamide as first-line systemic intensification therapy in the mHSPC setting were included. Subjects with insufficient clinical follow-up data or unavailable baseline laboratory parameters were excluded from the analysis. A flowchart illustrating patient selection, eligibility assessment, treatment allocation, and study analyses is presented in Figure 1.

Baseline demographic, clinical, and laboratory characteristics were retrospectively collected from medical records. These included age, Eastern Cooperative Oncology Group performance status (ECOG-PS), Gleason score, disease volume, presence of visceral metastasis, prostate-specific antigen (PSA), hemoglobin (Hgb), glomerular filtration rate (GFR), alkaline phosphatase (ALP), and lactate dehydrogenase (LDH). Disease volume was classified as low-volume or high-volume disease according to CHAARTED criteria [9]. Baseline laboratory values were obtained within 30 days before initiation of ARPI treatment. Cut-off values for Hgb, GFR, ALP, and LDH were selected according to commonly used clinical thresholds and institutional laboratory reference ranges. PSA was dichotomized using the cohort median value.

### 2.2. Study Endpoints

The primary endpoint of the study was rPFS, because it represents an established measure of disease control and treatment efficacy in advanced prostate cancer. Radiologic disease progression was evaluated using RECIST version 1.1 criteria [12]. Progression assessments were based on imaging studies obtained during routine patient management. The timing and modality of imaging examinations were determined by treating physicians according to clinical indications. Imaging assessments were performed using computed tomography, magnetic resonance imaging, bone scintigraphy, or positron emission tomography/computed tomography. As this was a retrospective real-world study, imaging evaluations were not scheduled according to a predefined study protocol but were performed at the treating physician’s discretion based on routine clinical practice, patient characteristics, and suspected disease progression. rPFS was defined as the time in months from initiation of abiraterone or enzalutamide treatment to radiographic progression or death from any cause, whichever occurred first. Patients without documented progression or death were censored at the date of last follow-up.

Exploratory subgroup analyses were performed according to predefined baseline clinical and laboratory parameters, including age (<70 vs. ≥70 years), ECOG-PS (0 vs. ≥1), Gleason score (<8 vs. ≥8), disease volume (low vs. high), visceral metastasis status, baseline PSA (<30 vs. ≥30 ng/mL), baseline Hgb (≥12 vs. <12 g/dL), baseline GFR (≥60 vs. <60 mL/min), baseline ALP (<147 vs. ≥147 U/L), and baseline LDH (<280 vs. ≥280 U/L).

### 2.3. Statistical Analysis

Categorical variables were summarized as frequencies and percentages. Group comparisons were performed using the chi-square test when expected cell counts were adequate; otherwise, Fisher’s exact test was applied. Continuous variables are presented as median values with corresponding ranges. Time-to-event analyses were performed using Kaplan–Meier survival estimates, and differences between groups were assessed with the log-rank test. Exploratory subgroup analyses examined rPFS outcomes according to predefined baseline clinical and laboratory characteristics in patients receiving abiraterone or enzalutamide. Relative risks of progression were quantified using univariable Cox proportional hazards regression models, and results are reported as hazard ratios (HRs) with 95% confidence intervals (CIs). Median rPFS values and progression event rates were also summarized for each subgroup. Statistical significance was defined as a two-sided *p*-value <0.05. All analyses were conducted using IBM SPSS Statistics version 27 (IBM Corp., Armonk, NY, USA).

## 3. Results

### 3.1. Patient Characteristics

The study cohort consisted of 172 patients with mHSPC who received first-line ARPI treatment in combination with ADT, including 84 treated with abiraterone and 88 treated with enzalutamide. Median follow-up for the overall population was 24.7 months (95% CI 21.5–27.9 months). Detailed baseline demographic and clinical characteristics are presented in Table 1.

The distribution of ECOG-PS, Gleason score, disease volume, visceral metastasis status, baseline PSA, Hgb, GFR, ALP, and LDH levels was generally comparable between treatment groups. Patients in the abiraterone group were significantly younger than those in the enzalutamide group, with a higher proportion of patients aged <70 years in the abiraterone arm (63% vs. 43%, *p* = 0.009). Except for age distribution, baseline characteristics were well balanced between treatment groups.

### 3.2. Radiographic Progression-Free Survival in the Overall Population

A total of 46 radiographic progression events occurred during the observation period. Progression was documented in 27 patients (32%) receiving abiraterone and 19 patients (22%) receiving enzalutamide. Median rPFS was 50 months in the abiraterone group and 49 months in the enzalutamide group, with no statistically significant difference between treatment strategies (*p* = 0.21).

### 3.3. Exploratory Subgroup Analyses

Exploratory subgroup analyses of rPFS according to baseline clinical and laboratory characteristics are presented in Table 2.

**Figure 2:**
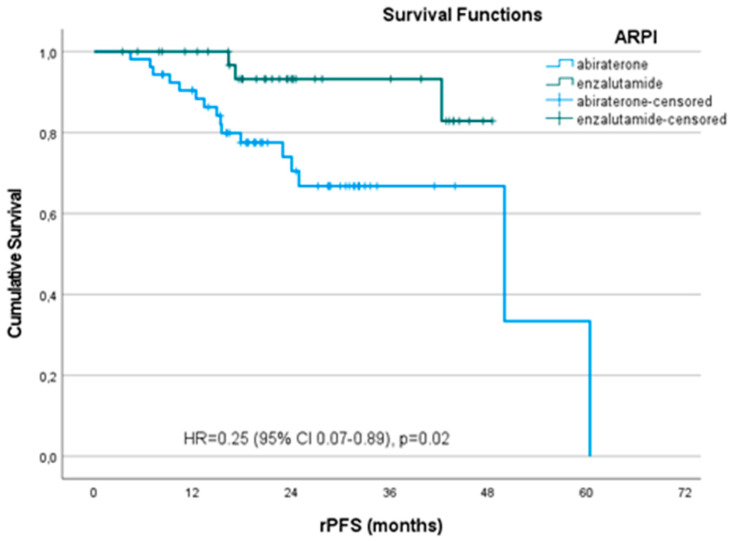
Kaplan–Meier curves for radiographic progression-free survival according to ARPI type in patients aged <70 years.

Within the subgroup of patients younger than 70 years, treatment with enzalutamide was associated with a significantly lower risk of radiographic progression compared with abiraterone (HR 0.25, 95% CI 0.07–0.89; *p* = 0.02) (Figure 2). No corresponding difference was observed among patients aged 70 years or older.

**Figure 3:**
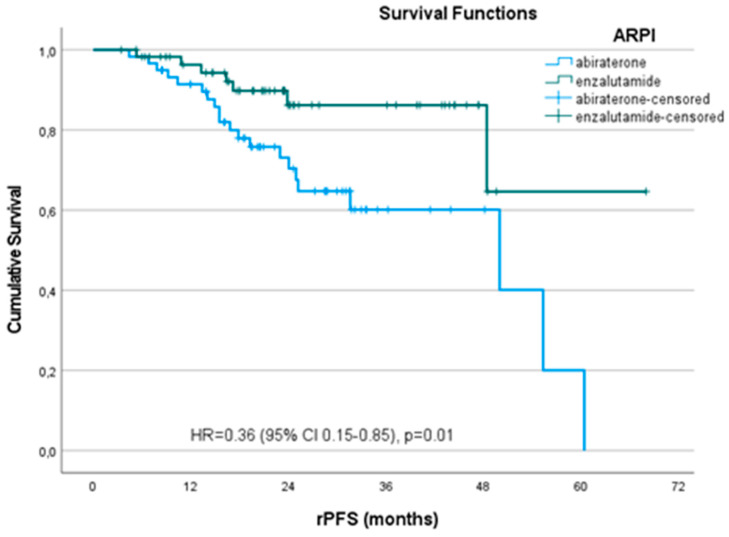
Kaplan–Meier curves for radiographic progression-free survival according to ARPI type in patients with baseline hemoglobin level ≥12 g/dL.

Among patients with baseline hemoglobin levels ≥12 g/dL, enzalutamide was associated with superior rPFS compared with abiraterone (HR 0.36, 95% CI 0.15–0.85; *p* = 0.01) (Figure 3). In contrast, outcomes did not differ significantly among patients with baseline hemoglobin levels <12 g/dL.

**Figure 4:**
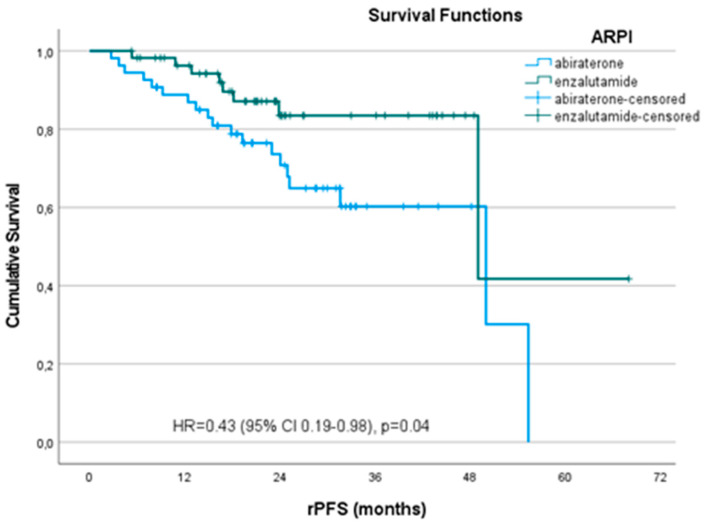
Kaplan–Meier curves for radiographic progression-free survival according to ARPI type in patients with baseline alkaline phosphatase level <147 U/L.

A significant difference in rPFS was also observed among patients with baseline ALP levels <147 U/L, favoring enzalutamide over abiraterone (HR 0.43, 95% CI 0.19–0.98; *p* = 0.04) (Figure 4). No significant treatment-related difference was identified in patients with higher baseline ALP levels.

No statistically significant differences in rPFS between treatment groups were observed according to ECOG-PS, Gleason score, disease volume, visceral metastasis status, baseline PSA level, baseline GFR, or baseline LDH level. Nevertheless, a non-significant trend favoring enzalutamide was observed among patients with baseline LDH < 280 U/L (HR 0.51, 95% CI 0.25–1.05; *p* = 0.06).

## 4. Discussion

In this retrospective real-world study, we evaluated rPFS in patients with mHSPC treated with first-line abiraterone or enzalutamide, with a particular focus on exploratory subgroup analyses. In the overall study population, rPFS outcomes were comparable between the two treatment groups. However, subgroup analyses suggested that enzalutamide may be associated with improved rPFS in selected patient subgroups, particularly among younger patients, patients with preserved baseline Hgb levels, and patients with lower baseline ALP levels.

Both abiraterone and enzalutamide have demonstrated substantial survival benefits in randomized clinical trials and are widely accepted standard treatment options in mHSPC [1,2,3,4,5]. Nevertheless, direct comparative evidence between these agents remains limited, particularly in routine clinical practice settings. As treatment selection is frequently individualized according to patient characteristics, comorbidities, disease burden, and physician preference, identification of baseline clinical factors potentially associated with differential treatment outcomes may have practical clinical relevance. In the present study, no statistically significant difference in rPFS was observed between treatment groups in the overall population. This finding is generally consistent with the absence of prospective head-to-head randomized comparisons demonstrating clear superiority of one ARPI over another in mHSPC. Although most available comparative data originate from metastatic castration-resistant prostate cancer populations, available comparative evidence, including systematic reviews and randomized comparative studies, has not demonstrated a consistent superiority of either abiraterone or enzalutamide across unselected patient populations [6,7]. However, our subgroup analyses demonstrated that enzalutamide was associated with significantly longer rPFS among patients aged <70 years, those with baseline Hgb ≥ 12 g/dL, and those with baseline ALP < 147 U/L. These findings may suggest that patients with more favorable baseline clinical and biological characteristics derive greater benefit from enzalutamide-based therapy.

Although the underlying mechanisms remain uncertain, several hypotheses may explain the observed subgroup differences. Younger age and preserved hemoglobin levels may reflect better overall functional status and lower systemic disease burden. Similarly, lower ALP levels are generally associated with reduced skeletal metastatic involvement and more favorable disease biology in advanced prostate cancer [8,9,10,11]. Collectively, these factors may identify a subgroup of patients with less aggressive disease and potentially greater responsiveness to androgen receptor-targeted therapies.

Enzalutamide directly inhibits androgen receptor signaling at multiple levels, including inhibition of androgen receptor nuclear translocation and DNA binding, whereas abiraterone primarily suppresses androgen biosynthesis through CYP17 inhibition [13,14]. It is conceivable that tumors characterized by lower disease burden and more favorable biological features remain more dependent on androgen receptor signaling and may therefore derive greater benefit from direct androgen receptor blockade with enzalutamide. Although a non-significant trend favoring enzalutamide was also observed among patients with lower baseline LDH levels, this finding did not reach statistical significance and should be interpreted cautiously. Nevertheless, these observations remain hypothesis-generating and require validation in larger prospective studies.

Our study has several important limitations. First, the retrospective single-center design introduces an inherent risk of selection bias and unmeasured confounding. Second, baseline age distribution differed significantly between treatment groups, with younger patients more frequently receiving abiraterone. As age was also identified as a significant subgroup variable, residual confounding cannot be excluded and may have influenced the observed subgroup findings. Third, imaging assessments were performed according to routine clinical practice rather than a predefined study protocol, which may have introduced variability in the timing of radiographic progression assessment. Finally, the relatively limited sample size, low number of progression events, and moderate follow-up duration reduced the statistical power of the study, particularly for subgroup analyses. Given the exploratory nature of the study and the limited number of events, interaction analyses, multivariable analyses, and propensity score-based adjustment methods were not performed in order to avoid potentially unstable or overfitted models. Therefore, the observed subgroup findings should be interpreted cautiously and considered hypothesis-generating.

Despite these limitations, our study provides real-world data regarding potential clinical factors associated with differential rPFS outcomes in patients receiving first-line ARPI therapy for mHSPC. To our knowledge, comparative real-world subgroup analyses focusing on routinely available baseline clinical and laboratory parameters remain limited in this setting. Larger multicenter studies with longer follow-up and prospective validation are warranted to confirm these exploratory findings and to better define patient subgroups that may derive differential benefit from specific ARPI strategies.

In conclusion, rPFS was comparable between abiraterone and enzalutamide in the overall mHSPC population. However, exploratory subgroup analyses suggested that younger age, preserved hemoglobin levels, and lower ALP levels may be associated with improved rPFS outcomes with enzalutamide. These findings raise the possibility that baseline clinical and laboratory characteristics may influence treatment outcomes in patients receiving first-line ARPI therapy. Nevertheless, the results should be interpreted cautiously, remain hypothesis-generating, and require validation in larger prospective studies.

## Figures and Tables

**Table 1:** Baseline characteristics of the patients (ALP: Alkaline phosphatase, ECOG-PS: Eastern Cooperative Oncology Group Performance Status, GFR: Glomerular filtration rate, Hgb: Hemoglobin, LDH: Lactate dehydrogenase, PSA: Prostate specific antigen).

Characteristic	Abiraterone (*n* = 84)	Enzalutamide (*n* = 88)	*p*-Value
Age (years), *n* (%)			**0.009**
<70	53 (63)	38 (43)	
≥70	31 (37)	50 (57)	
ECOG-PS, *n* (%)			0.73
0	48 (57)	48 (55)	
≥1	36 (43)	40 (45)	
Gleason score, *n* (%)			0.59
<8	24 (30)	22 (26)	
≥8	56 (70)	62 (74)	
Missing	4	4	
Disease volume, *n* (%)			0.09
Low	37 (44)	50 (57)	
High	47 (56)	38 (43)	
Baseline visceral metastasis, *n* (%)			0.47
No	68 (81)	74 (85)	
Yes	16 (19)	13 (15)	
Missing	0	1	
Baseline PSA (ng/mL), *n* (%)			0.09
<30	36 (44)	49 (57)	
≥30	46 (56)	37 (43)	
Missing	2	2	
Baseline Hgb (g/dL), *n* (%)			0.44
≥12	59 (81)	59 (76)	
<12	14 (19)	19 (24)	
Missing	11	10	
Baseline GFR (mL/min), *n* (%)			0.38
≥60	62 (85)	62 (79)	
<60	11 (15)	16 (11)	
Missing	11	10	
Baseline ALP (U/L), *n* (%)			0.86
<147	54 (78)	57 (77)	
≥147	15 (22)	17 (23)	
Missing	15	14	
Baseline LDH (U/L), *n* (%)			0.23
<280	54 (77)	62 (85)	
≥280	16 (23)	11 (15)	
Missing	14	15	

**Table 2:** Analysis of rPFS between abiraterone and enzalutamide in all patients and subgroups (ALP: Alkaline phosphatase, ECOG-PS: Eastern Cooperative Oncology Group Performance Status, GFR: Glomerular filtration rate, Hgb: Hemoglobin, LDH: Lactate dehydrogenase, NR: Not reached, PSA: Prostate specific antigen, rPFS: Radiographic progression-free survival).

Subgroup	Abiraterone (*n* = 84)		Enzalutamide (*n* = 88)		*p*-Value (Log-Rank)
	Event *n* (%)	Median rPFS (Months)	Event *n* (%)	Median rPFS (Months)	
All patients	27 (32)	50	19 (22)	49	0.21
Age (years)					
<70	16 (30)	50	3 (8)	NR	**0.02**
≥70	11 (35)	55.3	16 (32)	48.4	0.97
ECOG-PS					
0	13 (27)	50	10 (21)	NR	0.97
≥1	14 (39)	55.3	9 (22)	49	0.15
Gleason score					
<8	7 (29)	55.3	3 (14)	NR	0.53
≥8	16 (29)	50	16 (26)	49	0.60
Disease volume					
Low	10 (27)	50	5 (10)	49	0.18
High	17 (36)	55.3	14 (37)	48.4	0.98
Baseline visceral metastasis					
No	20 (29)	50	16 (22)	49	0.39
Yes	7 (44)	NR	3 (23)	NR	0.47
Baseline PSA (ng/mL)					
<30	12 (33)	50	6 (12)	49	0.08
≥30	15 (33)	55.3	13 (35)	48.4	0.89
Baseline Hgb (g/dL)					
≥12	21 (36)	50	7 (12)	NR	**0.01**
<12	5 (11)	NR	36 (58)	19.7	0.38
Baseline GFR (mL/min)					
≥60	23 (37)	50	13 (21)	49	0.28
<60	3 (27)	NR	5 (31)	48.4	0.90
Baseline ALP (U/L)					
<147	19 (35)	50	8 (14)	49	**0.04**
≥147	6 (40)	NR	10 (59)	19.7	0.51
Baseline LDH (U/L)					
<280	20 (63)	55.3	12 (19)	49	0.06
≥280	5 (31)	50	5 (45)	19.7	0.26

## Data Availability

The data presented in this study are available on reasonable request from the corresponding author. The data are not publicly available due to institutional and patient privacy restrictions.
